# Comparison of community-based HIV counselling and testing (CBCT) through index client tracing and other modalities: Outcomes in 13 South African high HIV prevalence districts by gender and age

**DOI:** 10.1371/journal.pone.0221215

**Published:** 2019-09-06

**Authors:** Simukai Shamu, Thato Farirai, Locadiah Kuwanda, Jean Slabbert, Geoffrey Guloba, Sikhulile Khupakonke, Suzanne Johnson, Nomea Masihleho, Julius Kamera, Nkhensani Nkhwashu

**Affiliations:** 1 Foundation for Professional Development, Health Systems Strengthening Division, Pretoria, South Africa; 2 University of the Witwatersrand, School of Public Health, Johannesburg, South Africa; 3 USAID Southern Africa, Pretoria, South Africa; University of Ghana College of Health Sciences, GHANA

## Abstract

**Background:**

To increase HIV case finding in a Community-based HIV counselling and testing (CBCT) programme, an index client tracing modality was implemented to target index clients’ sexual network and household members.

**Objective:**

To compare index client tracing modality’s outcomes with other CBCT recruitment modalities (mobile, workplace, homebased), 2015–2017.

**Methods:**

Trained HIV counsellors identified HIV positive clients either through offering HIV tests to children and sexual partners of an HIV index client, or randomly offering HIV tests to anyone available in the community (mobile, home-based or workplace). Socio-demographic information and test results were recorded. Descriptive comparisons of client HIV test uptake and positivity were conducted by method of recruitment—index client tracing vs non-targeted community outreach.

**Results:**

Of the 1 282 369 people who tested for HIV overall, the index modality tested 3.9% of them, 1.9% in year 1 and 6.0% in year 2. The index modality tested more females than males (55.8% vs 44.2%) overall and in each year; tested higher proportions of children than other modalities: 10.1% vs 2.6% among 1–4 years, 12.2% vs 2.6% among the 5–9 years and 9.6% vs 3.4% among the 10–15 years. The index modality identified higher HIV positivity proportions than other modalities overall (10.3% 95%CI 10.0–10.6 vs. 7.3% 95%CI 7.25–7.36), in year 1 (9.4%; 8.9–9.9 vs 6.5%; 6.45–6.57) and year 2 (10.6%; 10.3–10.9 vs 8.2%; 8.09–8.23). Higher proportions of females (7.5%;7.4–7.5) than males (5.5%;5.4–5.5) tested positive overall. Positivity increased by age up to 49y with year 2’s increased targeting of sexual partners. Overall linkage to care rose from 33.3% in year 1 to 78.9% in year 2.

**Conclusions:**

Index testing was less effective in reaching large numbers of clients, but more effective in reaching children and identifying HIV positive people than other modalities. Targeting HIV positive people’s partners and children increases HIV case finding.

## Introduction

The global HIV epidemic remains a major public health problem. In 2016 alone, at least 2 million people got infected with HIV mostly through heterosexual transmission[1]. Most of the infected people lived in sub-Saharan Africa. The diagnosis of people at risk of HIV infection has increased with new approaches added to HIV testing strategies, including home based, mobile, index, workplace testing and home self-test to reach more people who would otherwise be difficult to reach through provider-initiated testing at the health facility[2,3]. By 2015, up to 17 million people out of 36.7 million people living with HIV had been tested globally and were receiving treatment[1]. In view of the huge HIV testing gap, the UNAIDS introduced the ambitious 90-90-90 goals to test 90% of the HIV infected people by 2020[4]. However, up to 14.5 million, or 46%, of the people living with HIV did not know their HIV status by 2016[5–7]. South Africa had an estimated 7.1 million people living with HIV (PLHIV), with nearly 270 000 people newly infected in 2016[8,9]. Of these PLHIV, almost a quarter (23.7%) remained undiagnosed[10] with only four years before the UN target goals were expected to be reached.

In view of this testing gap targeted approaches are needed to increase HIV testing of HIV infected people. Assisted partner notification is an established approach used in public health to control the spread of communicable diseases through testing and treating one’s sexual partners and children [11]. It has successfully been used in sexually transmitted infections (STI)[11,12] and tuberculosis (TB)[13] control but has not been fully employed and evaluated in large community-based HIV counselling and testing (CBCT) programmes in South Africa. Through partner notification, children or sex partners of the individuals diagnosed with HIV (defined as index patients/clients) are informed of their risk of HIV infection due to their exposure to HIV infection and encouraged to test for HIV as well[14]. It also aims to prevent HIV reinfection and promote early linkage to care. Notification is usually done through the client, provider or both[15] as described below. Firstly, in simple patient referral the health service personnel advise index patients to disclose their HIV status to their partners and encourage them to test. However, as few as 11% notify their partners with between 3.5% and 14.6% experiencing a violent reaction thereafter[16]. Secondly, in the provider referral, third parties who are usually counsellors take the responsibility to notify the partners and this has higher uptake rate than simple patient referral[14]. Lastly, in contract referral (or conditional referral) a health provider agrees with the index patient that the latter will notify their partner but if that does not happen within a specified period the provider will notify the partner. Contract referral results in an increase in testing for STIs[14]. Emerging studies tested provider referral partner notification for HIV infections[17] and found it acceptable to clients, effective and feasible although in small scale studies[18]. More partners accept HIV testing and test HIV positive via assisted active partner notification than through passive referral and with fewer instances of partner violence[19]. Moderate to strong evidence in the feasibility, acceptability and safety of partner notification has led to the WHO recommending and issuing HIV guidelines for partner notification in 2016[19]. It is also proving to be effective in Sub-Saharan Africa[20–22] and with few cases of partner violence reported[15].

Evidence from these studies need demonstration outside of controlled research studies, with more improved strategies of notifying partners, to increase effectiveness, while also limiting side effects such as violence. The HIV index client tracing modality was designed to address the low HIV case finding in a South African CBCT programme. It was implemented to provide an opportunity for targeted HIV testing of the index client’s sexual network and household members considered to be at high risk of HIV infection[23]. The aim of this paper is to describe, using data from a CBCT programme implemented by the Foundation for Professional Development (FPD), the index client tracing modality’s outcomes (uptake and positivity) in comparison with other CBCT modalities implemented between October 2015 and September 2017 in 13 districts.

## Methods

**Study design:** A cross sectional study consisting of people of all ages was conducted in selected districts. Participants were offered HIV testing by trained HIV counsellors in communities where they lived or worked.

**Study setting:** The study was conducted in 13 South African districts across urban, rural, peri-urban, including informal settlements, farming and mining communities. The selected 13 districts were part of the 27 districts defined by the South African National Department of Health as high HIV prevalence districts which account for 82% of PLHIV in South Africa. PEPFAR funding was provided in these 13 districts to accelerate HIV testing and treatment between 2014 and 2018[24]. For example, seven of the 13 districts were from Gauteng and KwaZulu-Natal provinces which contribute 54% of total HIV burden in South Africa. At the start of the programme antenatal HIV prevalence in most of these implementation districts were above the national average (27%) and some districts recorded as high as 44% pregnant women with HIV[25]. The population in the districts were predominantly black, 52% were women, most of the residents belonged to the low socio-economic groups with low levels of education. Access to social amenities such as electricity, in-house piped water was low in these districts.

### The HIV index patient tracing modality

FPD implemented a CBCT programme in 13 high HIV burden districts across South Africa with funding from the PEPFAR grant[26] in 2014. The aim of the grant was to implement a programme to correctly identify people living with HIV (PLWH) and to effectively link them into HIV care and treatment programs. This was implemented in high HIV incident communities near where people live and work, complementing facility-based HIV Testing and Counselling services (HTS) and reaching HIV positive community members who may not otherwise access HTS in the health facility setting. FPD implemented the CBCT through four strategies of recruitment and testing termed modalities namely, systematic home-based HTS (door to door testing), index patient tracing HTS, mobile HTS and workplace HTS [23]. See Table 1 for these modalities’ characteristics. The realisation that those clients who were diagnosed with HIV needed assistance in disclosing their HIV status and that there was need to test their children and sexual network members at risk of HIV, led to the implementation of the Index client tracing modality in October 2015 through September 2017. The modality gave an important opportunity to test children together with their parent(s) in the index modality. In year 1 (October 2015-September 2016), the index modality recruited at risk household members and sexual partners and sexual network members for testing. In year 2 (October 2016-September 2017), mostly sexual partners who included main partners, and sexual network members were recruited and tested. This was done to focus the testing on the most at-risk people who in this case were the sexual partners as opposed to family members in general.

**Table 1:** Basic characteristics of the index tracing and other modalities.

	HIV testing Modality			
Description	Index tracing modality	Mobile modality	Mobile: Workplace modality	Home-based modality (door-to-door testing)
**Person targeted**	Sexual partners, sexual network members or family members (children)	General population	Workers at companies/factories	Household members
**Place**	Mostly homes	Shopping malls, public places, sporting events, taverns, etc	Workplaces	Homes
**Recruitment process**	Received contacts from an HIV positive person. Visit by appointment when possible but mostly concealed referral for anonymity	Set up testing tent/ gazebo in a community setting and invite passers by	Request employers’ permission. Visit by appointment.	Enter community households and test in homes
**Method of HIV testing**	Used South African algorithm	Used South African algorithm	Used South African algorithm	Used South African algorithm

**Fig 1:**
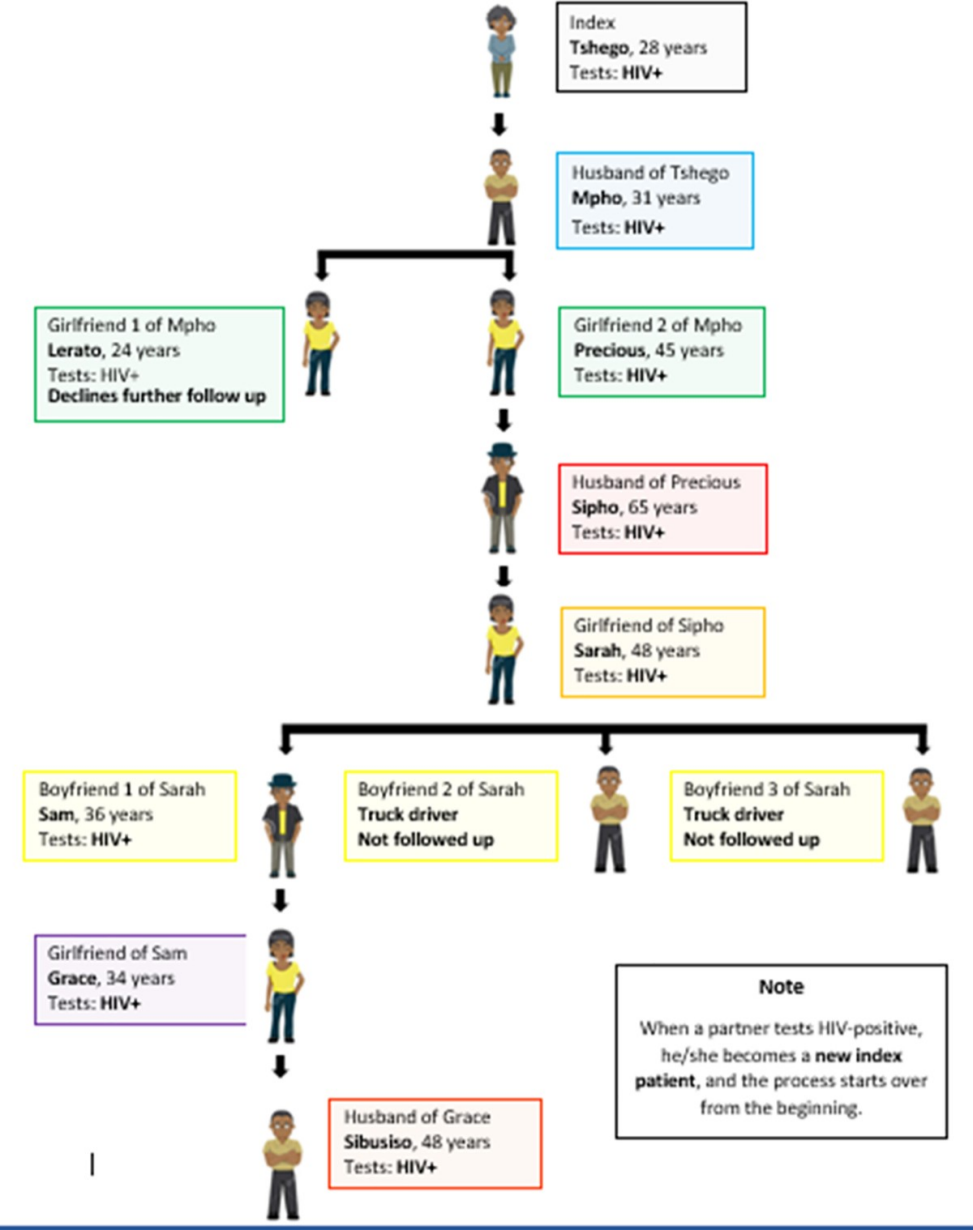
Diagrammatic sexual network mapping used to train index counsellors.

Fig 1 shows the process of index client tracing. Counsellors initially recruited HIV positive clients from the health facility’s active PMTCT, STI, ART and/or TB patients in the referral health facility or directly from the CBCT program. They requested and obtained consent from the index clients to visit their family members and sexual partners at home to offer them HTS. Clients tested for STI/HIV/TB were asked at the point of testing to consent to provide health workers access to information relevant for controlling the epidemic. Clients who were diagnosed HIV positive were then requested to be recruited as index patients. For those HIV positive clients who gave formal consent, the counsellors asked for contact information for all sex partners or the sexual network of the index client. With the index client’s permission, the counsellor visited the families and/or the sex network of the index client to provide HTS. Partners in the index client network who tested HIV positive become new index partners and were requested to name their other partners to be traced.

For clients who were not prepared to disclose their status to partners or their children, counsellors concealed the referral and planned nature of HIV testing from the particular partner/children so that it appeared random. Index client anonymity was further protected by testing at least two more surrounding households before and after visiting the family or sex network of the index client. Anonymising the referral by the index client protected the index client, minimised the blaming of the index client, including possibility of intimate partner violence, and helped to mitigate stigma as neighbouring homes were also offered HIV testing. Index tracing modality was advantageous because it increased staff productivity by testing surrounding households together with the index patient and linking index clients’ appointments for other households to test. HTS was conducted following the South African government HTS algorithm[26]. Those who tested HIV positive were either escorted, if they wanted, or referred to centrally accessible referral health facilities/point for care and usually assisted by making joint appointments (index client and partner/family) to ease linkage and further care.

**Study period:** The programme was implemented between October 2014 and October 2018. However, in this paper we only reported data collected from the 13 districts between October 2015 and September 2017 as data for the other years were not readily available for analysis. The analysis in this paper is therefore limited to this period.

**Study population:** All people aged 12 months or older living in the 13 defined districts formed the study population.

**Data collection and analysis:** Two primary outcome indicators were used to assess programme success, and these are HIV testing uptake and HIV positivity. Data for these variables were obtained through the routine information system designed for the programme during implementation. These indicators were documented on a secure programme database. HIV testing uptake was calculated based on the number of all clients identified, counselled and tested for HIV and included being offered a basic package of HIV-related services as necessary. HIV positivity was calculated as the total number of clients who tested HIV positive expressed as a percentage of the total number of clients tested. Linkage to care was calculated as the total number of HIV positive clients successfully linked to care and treatment in the programme year divided by the total number of all HIV positive clients multiplied by 100%. In year 2 of the programme, the definition of index tracing changed and so results were based mainly on sexual partners and sexual network members only. A questionnaire (see S1 Questionnaire) collected participants’ demographic characteristics such as age and gender through an interviewer administered questionnaire. De-identified data were cleaned and analysed descriptively, reporting percentages and confidence intervals (CIs). We compared index client data with data from other HTS modalities.

## Results

### HTS uptake by modality (index and other modalities)

Table 2 shows the numbers of clients tested by gender and age over the two-year period and disaggregated by testing modality and year of testing. A total of 1 282 369 clients tested for HIV during the two year period. Of these 660 351 tested in year 1 and 622 018 tested in year 2. Overall 3.9% clients tested through the index tracing modality compared to 96% who tested through other modalities. In year 1 and 2 1.9% vs 98.1% and 6.0% vs 94.0% of all clients tested through index testing and other modalities respectively. During the two-year period more females (55.8%) than males (44.2%) tested through the Index tracing modality. The index modality (44.2%: 43.8–44.7) tested fewer males than other modalities (46.7: 46.7–46.8) overall. Fewer males were tested through index modality in year 1 (42.1%) than year 2 (44.9%) while less women were tested in year 1 (55.1%) than in year 2 (57.9%). See Table 2.

**Table 2:** HIV testing proportions by modality and gender.

	TOTAL (Year 1 and 2)							Year 1					Year 2						
	Total			Other Modalities		Index		Total	Other Modalities		Index		Total			Other modalities		Index	
	N	%	95CI%	%	95CI%	%	95CI%	N	%	95CI%	%	95CI%	N	%	95CI%	%	95CI%	%	95CI%
**Total Tested by modality**	**1282369**	**100**	**-**	**96.1**	**96.1–96.2**	**3.9**	**3.8–3.9**	**660351**	**98.1**	**98.1–98.2**	**1.9**	**1.8–1.9**	**622018**	**100**	**-**	**94.0**	**93.9–94.0**	**6.0**	**6.0–6.1**
**Proportions of tested by gender**																			
Male	598078	46.6	46.5–46.7	46.7	46.7–46.8	44.2	43.8–44.7	306504	46.5	46.4–46.6	42.1	41.2–43.0	291574	46.9	46.8–47.0	47.0		44.9	44.4–45.5
Female	684291	53.4	53.3–53.4	53.3	53.2–53.4	55.8	55.3–56.2	353847	53.5	53.4–53.6	57.9	57.0–58.8	330444	53.1	53.0–53.2	53.0	52.9–53.1	55.1	54.6–55.6
**Total**	**1282369**	**100.0**	**-**	**100.0**	**-**	**100.0**	-	**660351**	**100.0**	**-**	**100.0**	-	**622018**	**100.0**	**-**	**100.0**	**-**	**100.0**	-

S1 Table shows HIV testing proportions by modality, gender and age. The index modality tested more children and young people compared to other modalities as follows: 1–4 years: 10.1% vs 2.6%; 5–9 years: 12.2% vs 2.6%; 10–14 years: 9.6% vs 3.4%. The index modality tested fewer proportions of adults than other CBCT modalities overall: 19.6%; 19.5–19.7 vs 13.0;12.7–13.3 among 20–24 year olds; 37.1%; 36.7–37.6 vs 49.5; 49.4–49.6 among the 25–49 year olds and 7.0%; 6.8–7.3 vs 9.6%: 9.6–9.7 among the 50 years or older age group. The same trend was observed in year 1 and year 2.

As shown on S1 Table the proportion of children tested through index tracing modality increased from year 1 to year 2 (8.5% to 10.6% among 1–4 year olds; 10.6% to 12.7% in 5–9 year olds and 8.8% to 9.9% in 10–14 year olds), while the proportion of adolescents tested decreased during the same period from 12.4% to 10.4% among 15–19 years, and 13.6% to 12.8% in 20–24 year olds. When data is disaggregated by gender, the index modality proportions of tested male clients increased significantly only among the 1–4 year olds from year 1 (8.5%;8.1–9.1) to year 2 (10.6%;10.3–10.9) but no significant change was observed in other modalities (2.7%; 2.6–2.7 in year 1 to 2.4%; 2.4–2.5 in year 2). There were decreases in the 50+ years in both the index modality (7.6%; 6.9–8.3 to 5.4%; 5.0–5.7) and other modalities (9.3%; 9.2–9.4 to 8.8%; 8.7–8.9). Significant increases in female testing proportions were observed in each of the 1–4 year, 5–9 year and 10–14 year age groups from year 1 to year 2 in the index modality while significant decreases were observed in the other modalities in the same age groups. There we no changes in the 20–24 year and 25–49 year in the index but a decrease and an increase were observed in the other modalities respectively. The 50+ year age group was characterised by a significant decrease in both the index and other modalities. See S1 Table for the levels of increase.

### HIV positivity

**Fig 2:**
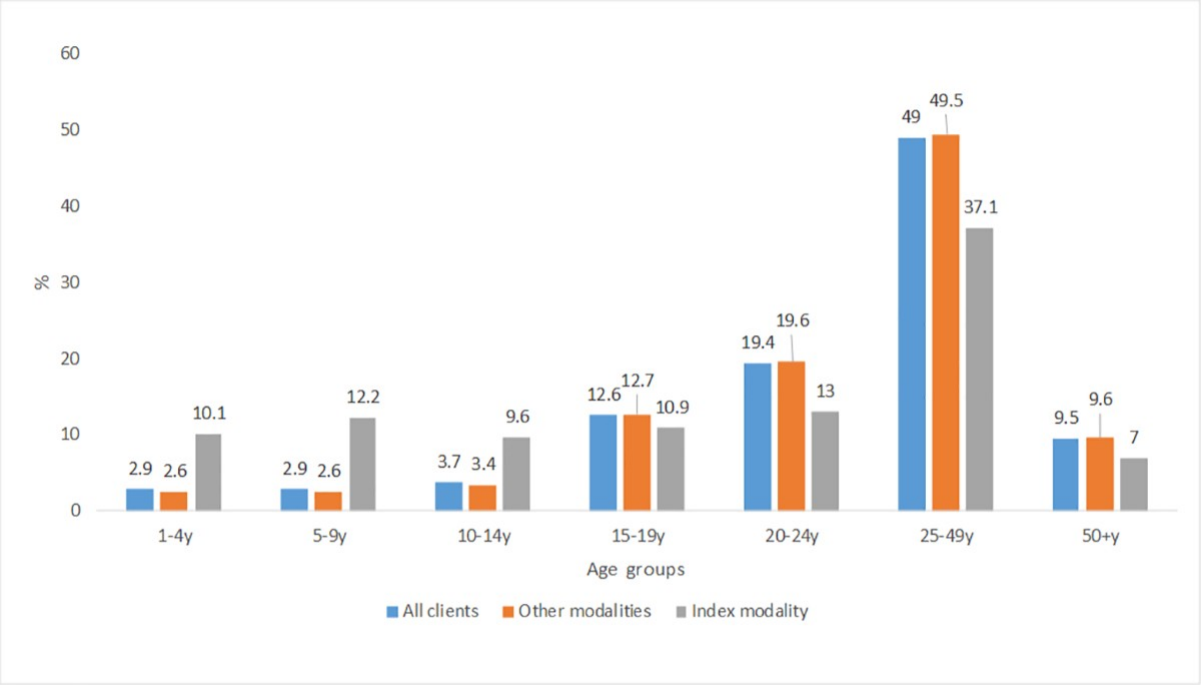
HIV positivity by age and modality.

**Fig 3:**
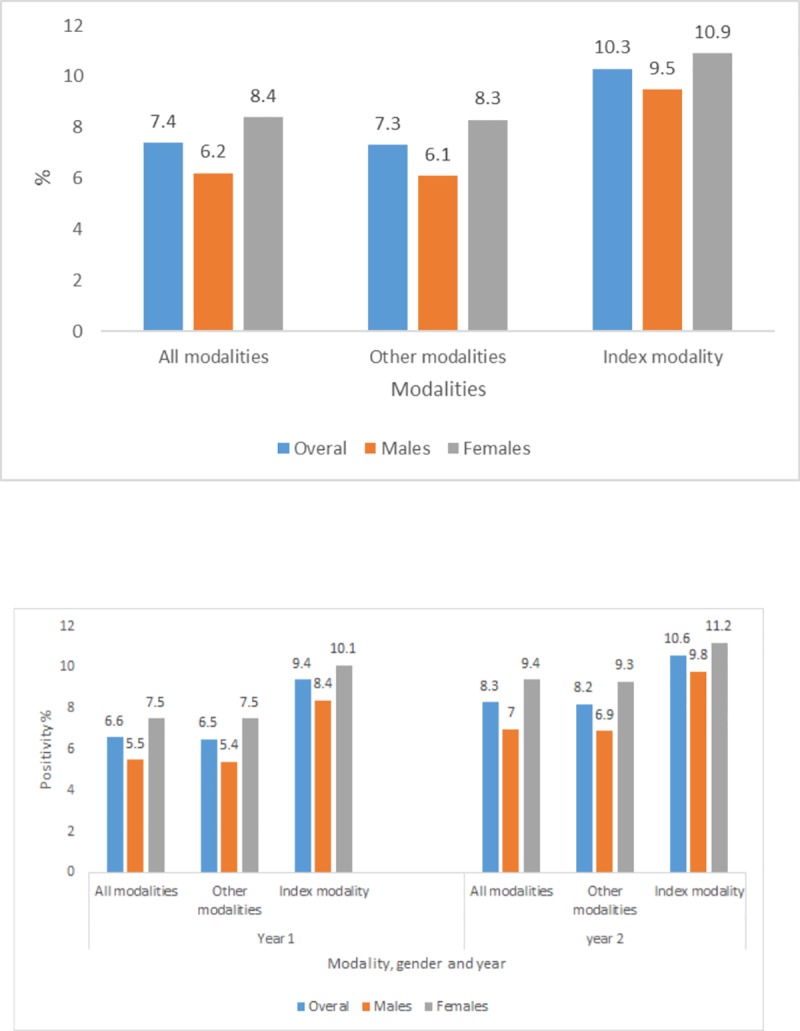
a. HIV positivity by modality, and b. HIV positivity by modality, gender and year.

Fig 2 shows HIV positivity by age group and modality while Fig 3A and 3B show HIV positivity by programme year, modality and gender. The overall positivity for the two years was 7.4% (6.6% in year 1 and 8.3% in year 2). The index modality overall recorded 10.3%; 10.0–10.6 compared to 7.3%; 7.3–7.4 in other CBCT modalities. In the index modality, positivity slightly increased from year 1 (9.4%; 8.9–9.9) to year 2 (10.6%; 10.3–10.9) compared to a relatively larger increase from 6.5%; 6.5–6.6 to 8.2%; 8.2–8.2 in the other modalities. Although the percentage increase was higher in other modalities than index modality, positivity remained higher in the latter. Higher positivity was found in females than in males overall (8.4% vs 6.2%) as well as in the Index modality than other modalities (10.9% vs 9.5%). Significant increases in HIV positive males were observed in both the index modality (from 8.4%; 7.7–9.2 in year 1 to 9.8%; 9.3–10.2 in year 2) and other modalities (from 5.4%; 5.3–5.5 to 6.9%; 6.8–7.0). The Index modality did not show any significant increases in the females’ positivity from year 1 (10.1%; 9.4–10.8) to year 2 (11.2%; 10.8–11.7) which the other modalities showed (from 7.5%; 7.4–7.5 in year 1 to 9.3%; 9.2–9.4 in year 2) See S2 Table.

S2 Table shows HIV positivity by programme year, modality, age and gender. As in other testing modalities, we found the positivity in the index client tracing modality increasing by age group up to age 49. Higher positivity was reported in the index modality than other modalities among the under-fives and all clients 20 years or older. HIV positivity differed by gender overall particularly among the index clients in the 20–24 years age group, where positivity among females nearly doubled (15.1%) that of their male counterparts (8.7%).

Due to the definition of the index client trailed changing to focus more on sexual partners than on sexual partners plus children of the index clients, an increase in the HIV positivity among the adults was demonstrated in both males and females while no significant increases were observed in the 1–19 year olds. An increase in positivity was shown in the 25–49 year olds from 15.5%; 14.5–16.6 to 19.0%; 18.3–19.6 and in the 50+ age group from 9.4%; 7.8–11.2 to 13.8%; 12.4–15.3. Huge increases were also observed in the male group in which positivity rose in the 20–24 year from 5.3%; 3.7–7.4 to 9.7%; 8.5–11.1. See S2 Table.

Overall linkage to care and treatment during this period was 56.1%. Programme year 1 linkage to care was 33.3%. The rate rose sharply to 78.9% in programme year 2. Data were not however disaggregated by modality and cannot be reported beyond these overall percentages.

## Discussion

The HIV outcomes of the index client tracing modality demonstrate that it is feasible to locate contacts of index clients’ family members, partners and people in their sexual network and test them for HIV. The outcomes of the study are encouraging for the HIV testing efforts. The index modality tested a relatively high proportion of men, although less than females, against a background that men were generally hard to reach with HIV testing. The index modality tested a lower proportion of children than adults since it targeted more sexual partners than family members during the period of analysis. Nevertheless, the analysis demonstrated that when compared to other modalities, the index modality was effective in targeting children (1–14 years) and young people (15–19 years) for testing. The rest of the population (20+ years) were identified more with other modalities than the index modality. The study found that although it identifies less people for testing overall as it targeted people who were sexually related to the index client, the index modality was effective in improving HIV case finding given its approach of targeted HIV testing. The study also demonstrated that as the index modality definition was narrowed to include sexual partners only positivity increased among adults. The index tracing modality is therefore effective in identifying more HIV positive sexual partners. We discuss these findings below.

Our study demonstrates a success story of a provider-assisted notification approach which involved the provider visiting and testing people at their homes with the permission of the index client. In most of the studies on assisted partner notification HIV testing does not take place at the home of the client. For example, prior studies included sending notification letters home or visiting target clients home to invite them to the clinic [27] which left room for the target client to refuse to attend the clinic[28]. In our study the client’s children and partners were visited and tested them in their homes. The programme cut down costs associated with clients’ travel to receive HIV testing and minimised chances of refusing to test as is the case if testing was based on simple client referral. The provider notification provides an opportunity to counsel the partner to prevent further infections and encourage linkage to care and has also been found to be relatively cost-effective in preventing new infections[28].

The index modality was effective in targeting high HIV-risk people with testing through the use of referrals provided by the index client unlike randomly testing all people in a given community. Available evidence also suggests that targeting populations or specific individuals maximises opportunities for testing HIV positive people[29]. Through the index approach the CBCT programme tested people who would not have been tested without negotiating challenges of disclosure as done in the index tracing process. Our results add to the existing findings that expedited or assisted partner notification significantly increases the proportion of partners tested[17,18,27,28] where partners were reluctant, unwilling or unable to test for various reasons. Earlier, Landis et al found that leaving the notification process to the index client is less effective for many reasons including the inability to convince a partner to test for HIV and the fear of violence[27]. Assisting the client to link his/her partner or children with counsellors helps to break the perceived and real barriers to uptake of HIV testing.

Our analysis demonstrated that index partners are useful in facilitating identification of sexual partners who are most likely to be HIV positive. This is because the index client most likely knows or highly suspects partners who infected them or were infected by them. This is especially clear from the extremely high positivity results in programme year 2 where index tracing recruitment and testing focussed on sexual partners. A previous study found that involving a partner had a threefold increase in uptake of care services and a fourfold increase in uptake of prevention services such as condom use[30]. Given that HIV is sexually transmitted, it is important to involve partners to identify their sexual partners who are at risk of HIV infection and assist linking them for care.

The study found that when compared to other modalities, the index modality was effective in testing children (1–14 years) and young people (15–19 years). Antenatal clinic registers of HIV positive index client mothers may have easily facilitated reaching a significant proportion of children. The low positivity of HIV among the under 5s in the index modality may have been influenced by the fact that the women who tested in antenatal clinics were more likely to have participated in the prevention of HIV transmission from mother to child resulting in more HIV negative tests among children in our programme. This proves the point that targeting children in the family of the index client is feasible but not highly effective in reaching HIV positive children in a country where vertical transmission has significantly been reduced to close to 2% in South Africa[31,32]. Further research would need to stratify results by where the index patients were enrolled for tracing (ante/postnatal health facility or CBCT programme) to validate our argument.

Although we found lower uptake among men than women, having 43.5% men in the total sample of index recruited participants is a huge leap towards closing the gender gap in HIV testing. This is a huge success in a programme and country where men are hard to reach for HIV testing[33,34]. Despite women’s gender specific challenges to successful partner notification [35,36] higher proportions of women still tested for HIV. Generally women have better health seeking behaviours than men and have additional opportunities during antenatal care for HIV testing which men do not have. However, a more detailed study is needed to assess how gender relations are barriers to partner notification outside of the well documented intimate partner violence barrier. More investment in testing men is needed to keep increasing numbers of men who know their HIV status.

Since one of the challenges of partner notification is the fear of intimate partner violence and the fear of disclosing one’s HIV positive status our programme anonymised the referral nature of the index testing unless when it was necessary and allowed by the index client. This technique enabled the programme to approach the client’s partners, sexual networks and families without the index client being victimised or blamed for HIV infection or for bringing the counsellors in their homes. It also facilitated uptake of HIV testing. Although an economic analysis is still to be conducted, index client testing appear to be cost-effective in identifying HIV positive clients and targeting children because it is focussed on certain individuals following the contact details provided by the index client. In addition, since it was implemented alongside other modalities, human resources were effectively used in the programme through different modalities for one goal–to increase uptake of HIV testing and to find the HIV positive clients.

Regarding linkage to care and treatment, the rise in the rates from year 1 to year 2 from 33.3% to 78.9% was not only attributable to the success in rolling out the index client modality. Several other factors contributed and these include relationship management between the CBCT programme and health facilities, provision of client escort services to facilities, addressing barriers at the health facility level including task shifting of administrative duties to non-clinical staff. Some of the successes in linkage to care and treatment in this programme were reported before[37].

The study has several limitations which must be discussed to assist the interpretation of the results. Firstly, the analysis relied on available programmatic data. This data was not specifically collected to answer our research question. As a result, we do not know if there were any partners who refused to share contacts with programme counsellors to contact partners or their children, nor do we know if there were partners or children of the index client who refused testing. Such data would have given us a sense of the acceptability of the index referral system as well as the extent of the utility of the index modality. Assessing linkage to care by modality would have helped to show the index modality’s contribution to the second 90 of the UN 90-90-90 goals. However, the programme’s linkage to care data were not disaggregated by testing modality. The study did not collect individual client data on possible harm (partner or family violence) associated with index testing. However, we learnt from the discussions with the programme staff that no harm was reported to them after offering HIV testing. Lastly, the data was not disaggregated at individual level and therefore limited our analysis to only descriptive and comparative analysis.

Despite these limitations the study has its strengths. Firstly, while prior studies on index client testing focussed on people defined in HIV terms as key populations[28,38] this study offers a community and population based perspective of index client testing in low resource settings in South Africa. Secondly, the study boasts a large sample drawn from 13 of the 27 high HIV prevalence districts in South Africa which is useful in demonstrating coverage and scalability of the approach. Thirdly, the approach used was built on decades of existing practical experiences in the control of communicable diseases such as TB and STI. We designed and implemented the programme beyond the previously practised partner notification methods in TB and STI research to assist protecting against possible harm while ensuring client confidentiality and high HIV uptake across age groups and gender in different communities. Lastly, we were able to demonstrate that it is feasible to identify HIV positive partners or family members through tracing recently tested HIV positive clients.

## Conclusions

The study aimed to describe the index modality’s outcomes. We highlighted that it was feasible to locate HIV positive clients’ family, sexual partners and sexual networks and test them for HIV. We also demonstrated that this method was effective in targeting children for testing and identifying HIV positive sexual partners and those in the index client’s sexual network, compared to other CBCT modalities. We therefore strongly recommend using the index client tracing modality to reach HIV positive clients in communities and hence significantly contribute to breaking the chain of HIV transmissions. Since the index modality relies on obtaining index clients from the newly diagnosed clients through CBCT and facility testing including those already on ART and are regularly visiting the health facilities to replenish their medication, we recommend that this method be used to complement other methods in the CBCT programme to target partners and those in the sexual network of the index partner for high positivity.

## Supporting information

S1 Questionnaire (PDF)

S1 Table HIV testing proportions by modality, year, gender and age group. (XLSX)

S2 Table HIV positivity by modality, year, gender and age group. (XLSX)
