# Supplementary material for: Comparison of community-based HIV counselling and testing (CBCT) through index client tracing and other modalities: Outcomes in 13 South African high HIV prevalence districts by gender and age
Source: PLoS One. 2019 Sep 6;14(9):e0221215. doi: 10.1371/journal.pone.0221215 (PMC6730921; doi:10.1371/journal.pone.0221215)
Supplement: S1 Questionnaire — (PDF) [file pone.0221215.s001.pdf]

Adult INFORMED CONSENT for HIV COUNSELLING & TESTING SERVICES

CLIENT INFORMATION																
**Testing Modality**	*HBHTS*		*MBHTS*		*WP-HTS*		*Twilight HTS*		*Index HTS*		**Test Type:**	*Individual*		*Couple*		*Family*
**Client Name:**	**Name:**							**Surname:**								
**ID Number:**																
Sex:	*M*	*F*	***Age***			**Testing place:**	***District***		***Sub-district***		***Testing site name***					
**Date of test**	*D*	*D*	*M*	*M*	*Y*	*Y*	*Y*	*Y*	**Rise member:**	*Y*	*N*	**Name of club/ school**		**Card No**		

I Hereby give signed consent to: (Tick Yes or No)

Yes	***No***	
☐	☐	**Be tested for HIV including CD4**
☐	☐	**A home visit by a Health Care Worker who will provide HCT to my family**
☐	☐	**Allow FPD to verify that i have reached the referral facility**
☐	☐	**Be contacted telephonically from FPD's call centre on any of the numbers supplied below.**

☐ **Cellular phone no:** ☐☐☐ ☐☐☐ ☐☐☐

☐ **Alternative number:** ☐☐☐ ☐☐☐ ☐☐☐

I have been informed and I have understood that: (My counsellor has explained the following)

- **All my test results and health information will be handled with strict confidence and will only be shared with reputable health care providers in the best interest of my health**
- **All topics discussed before, during and after the session, as well as during the process of Data Management, are equally treated as confidential.**
- **I understand, and have given my consent that the data might be used for reseach without disclosing my name or personal information.**

____

Signature: Client

____

Signature: HCT Provider

Right Thumb Print: Client

____

Date

HBHTS=Homebased HTS,MBHTS= Mobile HTS, WP HTS=Workplace HTS

Sponsored by

USAID FROM THE AMERICAN PEOPLE

SOUTH AFRICANS AND AMERICANS IN PARTNERSHIP TO FIGHT HIV/AIDS

A project of

FOUNDATION FOR PROFESSIONAL DEVELOPMENT FPD

First Ever test	Y	N	Date of last test	< 6 months		6 - 12 months		13 - 24 months		> 2 years	
Result of last HIV test	Negative		Positive		Don't Know						

TB Screening

Yes	*No*	*Have you had/been:*
		Cough(ing) for longer than 2 weeks
		Coughing up blood in your sputum
		Sudden unintentional/unexplained weight loss
		Night sweats for longer than 2 weeks
		Fever for longer than 2 weeks
		in close contact with someone who has been coughing for more than 2 weeks

STI Screening

Yes	*No*	*Did/ Do you have:*
		unprotected sex with HIV and STI suspect
		abnormal or smelly discharge from vagina or penis
		abnormal bleeding from vagina or penis
		experiencing pain during sex / when passing urine
		experiencing pain during sex / when passing urine

Pregnancy status

Yes	*No*	*Have you:*
		Missed your last normal periods
		Been using any form contraception
		Had unprotected sex in the last 3 months

Screened for non communicable diseases

Yes	*No*		*Results*
		Blood sugar	
		Blood pressure	
		PAP smear	Normal | Abnormal

Contraceptive Offered

Pill		*IUD*		*Implants*	

Circumcission

Yes	*No*	
		Are you circumcised

HIV Screening test:

Name of test		Lot No.		Expiry date		Test Result	Non reactive		Reactive		Invalid	

HIV Confirmatory

Name of test		Lot No.		Expiry date		Test Result	Non reactive		Reactive		Invalid	

Final Results

Nagetive		Positive		Indeterminate	

Couple final results

Cord neg		Cord pos		Discord	

Post counselled	Y	N

Key populations	Sex worker		MSM		IDU		Client of sex worker	

Known positive	Y	N	Ever on ART	Y	N	Comment	

Number of Condoms distributed

Male		Female	

Number of Lubricants distributed

Lubricants	

Referrals	VMMC		ANC		GBV		Pre PEP		TB		STI		Family Planning		ART		Social Services	
Other prevention services:																		

Referral Facility	

Index consent	Y	N

HTS reg No.		Page No.		Client row No.	

Sponsored by

USAID
FROM THE AMERICAN PEOPLE

SOUTH AFRICANS AND AMERICANS IN PARTNERSHIP TO FIGHT HIV/AIDS

A Project of

FOUNDATION FOR PROFESSIONAL DEVELOPMENT FPD
